# On the Influence of Pregnancy and the Puerperal State on the Progress of Phthisis

**Published:** 1852-11

**Authors:** 


From the Bulletin de l' Acad. Rev. Med.

*On the Influence of Pregnancy and the Puerperal State on the Progress of Phthisis, by* M. M. Grisolle and Dubreuilh.

M. Grisolle, in reporting to the Academy of Medicine upon a memoir presented by M. Dubreuilh, observes, that the views he formerly expressed have only obtained additional confirmation. In none of the thirteen cases related by M. Dubreuilh, or in the thirty-five now collected by M. Grisolle, has the power formerly vaguely attributed to pregnancy, of staying the progress of phthisis, been observed. In some cases, indeed, it seems to have played the part of determining cause, and in others to have aggravated the condition. According to M. Grisolle's observation, cases in which the first symptoms of phthisis are developed at an early period of pregnancy, and amidst a state of health otherwise satisfactory, are more common than those in which the pregnancy is consecutive to the early appearance of the organic disease. Both observers are, indeed, of opinion that phthisical women conceive with difficulty; and M. Delafond assured the reporter that cows, even at an early period of the disease, usually remained sterile, even though they continued fully alive to the attractions of the bull. He added, also, that in such as did conceive, abortion was common about the fifth or sixth month; while in such as went their full time, the progress of the disease was nowise modified. In M. Grisolle's former papers, he stated that pregnancy, in his cases, so far from retarding, hastened the progress of phthisis; and although the rate was found to be somewhat slow in M. Dubreuilh's cases, this probably occurred from their having arisen in private practice, while M. Grisolle's were all hospital patients. Both sets of cases, however, amply disprove the suspending power of pregnancy; and M. P. Dubois' experience has long since led him to a similar conclusion. Phthisis which has appeared at an early period of pregnancy, pursues a constantly onward course; and if improvement is to take place at all, it never does so until after delivery. It is rare for phthisis thus complicated to present those intermissions or sudden suspensions of progress sometimes met with in ordinary phthisis. The children brought forth by phthisical mothers, though usually small, are plump and well-looking to an extent that would not, *a priori*, be expected from persons suffering from so exhausting a disease.

M. Dubreuilh expresses a theoretical opinion in favor of the prevalent belief that the progress of phthisis is hastened by *delivery*, but his facts are against him; and so complete is the suspension of the disease sometimes, that delusive hopes of cure are entertained.

In regard to the influence of *phthisis on pregnancy*, both observers are agreed that such patients *ordinarily* go their full time; which must be regarded as a remarkable fact, when it is considered that more than one half of the pregnant women who are attacked by pneumonia, abort. Both also find that these women have very easy labors—a fact due to the smaller size of the child, and the relaxed state of the tissues. Both, too, consider that the attempt to suckle exerts the most disastrous influence on both mother and child.

